# Prevalence and associated factors of anemia among adult human immune deficiency virus positive patients on anti-retroviral therapy at Debre tabor Hospital, Northwest Ethiopia

**DOI:** 10.1186/s13104-019-4214-3

**Published:** 2019-03-25

**Authors:** Kassaw Wubneh Zerihun, Gashaw Andargie Bikis, Esmael Ali Muhammad

**Affiliations:** 1 Debre Tabor Health Science College, P.O. Box 196, Gondar, Ethiopia; 2 Department of Health Service Management, Institute of Public Health, University of Gondar, P.O. Box 196, Gondar, Ethiopia; 3 Department of Human Nutrition, Institute of Public Health, College of Medicine and Health Sciences, University of Gondar, P.O. Box 196, Gondar, Ethiopia

**Keywords:** Anemia, HIV, ART, Deber Tabor, Ethiopia

## Abstract

**Objective:**

Anemia is the most common hematological complication of HIV infection that has a significant impact on the quality of life and clinical outcomes. Therefore, the aim of this study was to assess the prevalence and associated factors of anemia among adult HIV positive patients on Anti-retroviral therapy at Deber Tabor hospital, northwest Ethiopia. An institution based cross-sectional study was conducted on 365 HIV/AIDS patients on ART selected using the systematic random sampling technique. Blood samples were analyzed using the Cell-DYN 1800 automated hematology analyzer to measure hemoglobin. Bivariable and multivariable binary logistic regression analyzes were employed to find the predictors at p-value < 0 .2 and 0.05, respectively.

**Results:**

The overall prevalence of anemia was 34.0%[95% CI (29.0, 39.0)]; taking Zidovudine based antiretroviral regimen (AOR: 5.9, 95% CI 1.04, 13.86), CD4 count < 200 cells/mm^3^ (AOR: 4.8 95%, CI 1.14, 12.42), inability to read and write (AOR: 3.2, 95% CI (1.24,8.40), inadequate dietary diversity (AOR: 2.2, 95% CI 1.15, 4.26), and female sex (AOR: 1.9, 95% CI 1.06, 3.69) were significantly associated with increased odds of anemia. Therefore, routine screening of hemoglobin level, proper treatment of respondents on zidovudine based ART regimen and increasing productivity to improve dietary diversity are essential to prevent anemia.

## Introduction

Anemia is a nutritional disorder resulting when the number and size of red blood cells or hemoglobin concentration falls below the cut-off value, consequently impairing the capacity of the blood to transport oxygen to the body [1]. It is among the extensively spread global public health problems, affecting both developing and well-developed countries with an impact on health and socio-economic development of nations [2].Hematological complications have been documented to be the second most common causes of morbidity and mortality among HIV sero-positive patients with a significant impact on quality of life and clinical outcomes [3–5].

The most common cause of anemia worldwide is iron deficiency, resulting from prolonged negative iron balance, caused by inadequate dietary iron intake, absorption, and other nutritional deficiencies like folic acid and vitamins B12. In addition to the above causes among HIV/AIDS patients, anemia could be associated with a highly active antiretroviral therapy (HAART) like zidovudine based regimen and opportunistic infections [6, 7].

Anemia is a common feature of HIV infection, occurring in about 35% of HIV/AIDS the patients who start antiretroviral treatment (ART) in Europe and North America [8], while a comprehensive figure for the burden of anemia in HIV/AIDS patients in Sub Saharan Africa hasn’t been estimated. There variation in the prevalence of anemia among HIV/AIDS patients ranges from 10.1% to 77.4% [2], whereas in local areas, particularly in southwest and eastern Ethiopia, its magnitude is 41.2% and 69.6%, respectively [2, 9]. It has been shown that anemia influences the natural history of HIV disease by accelerating the rate of disease progression and operating as a strong independent predictor of death [10–12]. Uncorrected anemia results in a multisystem disabling symptoms like fatigue, exhaustion, increased risk of HIV dementia, poor quality of life, decreased survival, and possibly even the exacerbation of poverty in countries, like Ethiopia where the prevalence of HIV/AIDS is high [3, 13].

In addition to variation its variability in magnitude, anemia is influenced by factors which are associated with adult HIV/AIDS patients on ART, socio demographic factors, ART regimen, and CD4 count [14–16]. Furthermore, there is paucity of information on the prevalence and associated factors of anemia among HIV/AIDS patients on ART in Ethiopia. Therefore, this study aimed to assess the prevalence and associated factors of anemia among adult HIV/AIDS patients on ART in Debre Tabor, hospital, northwest Ethiopia.

## Main text

### Methods

#### Study design and setting

An institution laboratory based cross-sectional study was conducted from March 3 to April 3, 2017. It was conducted in Debre Tabor hospital located 667 km from Addis Ababa, the capital of Ethiopia. The hospital has 253 health professionals. From the beginning, there were 4400 HIV positive patients enrolled at the ART clinic; on average, 45 HIV positive patients attended the ART clinic per day. At the moment, there were 1980 patients on ART 1822 of whom were adults  ≥ 18 years old.

#### Sample size and sampling procedure

To determine the sample size, we used the single population proportion formula by considering the following assumptions: a 95% confidence interval, 5% margin of error, 69% proportion of anemia [17] plus a 10% non response rate yielded the final sample size of 365. The participants were selected by the systematic sampling technique. According to the data from the hospital, the average monthly number of clients that attended in ART clinic was 990. The sampling fraction (kth) value was determined by dividing the total monthly clients by sample size (990/365 = 3), and the first respondent was selected by the lottery method.

#### Data collection and analysis

Data was collected through a face to face interview, using a structured and pre-tested questionnaire. For the measurement of hemoglobin level, venous blood was drown from each participant in a volume of 4 ml in ethylenediaminetetraacetic acid (EDTA) vacutainer tube. All specimens were properly labeled with patient codes. Hematological parameters were determined using a Cell-DYN 1800 automated hematology analyzer (Abbot Laboratories Diagnostics Division, USA), while CD4+ T cell count was collected from patient charts.

In order to maintain the quality of data, training was provided to data collectors and supervisors for two days by the principal investigator. A pretest was conducted on 5% of the subjects at Debre Tabor health center. On-site supervision was performed and each copy of the questionnaire was checked for completeness and accuracy before data entry. Standard Operating Procedures (SOPs) were used according to the manufacturer’s instructions for laboratory tests. Results of hemoglobin level testing were categorized to determine anemia [(hemoglobin level < 130 g/l for men > 15 years) and < 120 g/l for non pregnant women > 15 years)] [18]. Concerning the dietary diversity score, participants who consumed five or more food groups in the preceding 24 h were classified as meeting the minimum dietary diversity [19].

Data was cleaned, coded, and entered into Epi-info statistical software Version 7 and then transferred to SPSS version 20 for further analysis. Associations between dependent and independent variables were assessed by using the binary logistic regression, and variables with p values <0 .2 were entered into the multivariable logistic regression with a 95% Confidence Intervals. The corresponding p value of < 0.05 was considered as statistically significant at a 95% confidence interval.

### Results

#### Socio-demographic and health characteristics

In the study, a total of 365 HIV+ patients on ART were included with a response rate of 100%. One hundred thirty-nine (38.1**%**) of the participants were male; 162 (44.4%) were married. The mean age of the participants was 39.26 years with (St.d ± 10.88); 325 (89%) of the participants were urban dwellers. One hundred seventy seven (48.5%) were taking zidovudine containing ART regimen, and one-fifth (22.2%) were underweight, while one-third of the study participants (34%) had adequate dietary diversity (Table 1).

**Table 1:** Socio-demographic and health characteristics of HIV+ patients on ART at Debere Tabor Hospital, Northwest Ethiopia, 2017 (n = 365)

Variables	Frequency	Percent (%)
Sex		
Male	139	38.1
Female	226	61.9
Age		
18–30	80	21.9
31–45	198	54.2
46–60	77	21.1
> 60	10	2.7
Marital status		
Single	54	14.8
Married	162	44.4
Divorced	109	29.9
Widowed	40	11.0
Residence		
Urban	325	89.0
Rural	40	11.0
Educational status		
Unable to read and write	121	33.2
Able to read and write	45	14.8
Primary school	91	24.9
Secondary school	54	12.3
Diploma and above	54	33.2
Occupations		
Governmental	55	15.2
Privet	13	3.6
Unemployed	37	10.1
Agriculture	37	10.1
Merchants	72	19.7
House wife	78	21.4
Daily labor	73	20
WHO clinical stage		
Stage I	211	57.8
Stage II	70	19.2
Stage III	51	14.0
Stage IV	33	9.0
CD4-T cell count (cells/mm^3^)		
< 200	54	14.8
200–350	59	16.2
351–500	61	16.7
≥ 500	191	52.3
ART regimens		
AZT based regimen	177	48.5
TDF based regimen	173	47.4
Second line regimen	15	4.1
Duration of HAART Intake (months)		
3–30	65	17.8
31–60	88	24.1
61–90	102	27.9
91–120	87	23.8
> 120	23	6.3
Body mass index (kg/m^2^)		
< 18.5	83	22.7
18.5–24.9	255	69.9
≥ 25	27	7.4
Dietary diversity score		
Adequate dietary diversity	124	34.0
Inadequate dietary diversity	241	66.0
Other drugs intake		
Yes	112	30.7
No	253	69.3
Alcohol intake		
Yes	83	22.7
No	282	77.3

#### Association of anemia with body mass index (BMI)

**Fig. 1:**
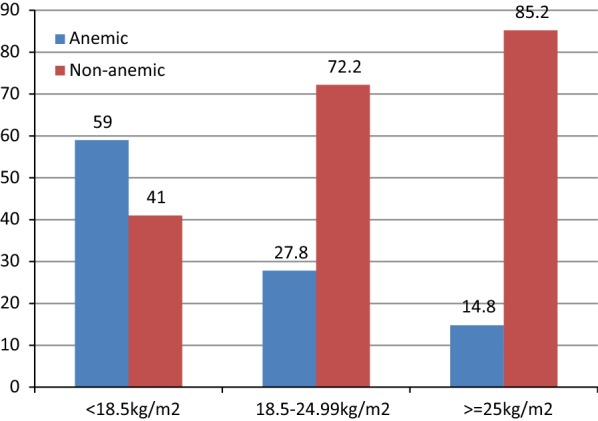
Anemia status by body mass index among HIV+ patients on ART at Debere Tabor Hospital, North West Ethiopia, 2017 (n = 365)

Over half, 215 (59%), of the participants who had less than 18.5 kg/m^2^ BMI were anemic, while only 54 (14.8%) of those whose BMI was ≥ 25 kg/m^2^ were so (Fig. 1).

#### Prevalence of anemia among HIV positive patients on ART

Participant Hgb level was used to determine the prevalence of anemia which turned out to be 124 (34%). Of those 23 (6.3%) and 101 (27.7%) were moderate and mild, respectively. The mean hemoglobin level of the participants was 13.1 (St.d ± 1.72).

#### Factors associated with anemia among HIV positive patients on ART

In multivariable analysis; ART regimen, CD4 count, dietary diversity, educational status, and sex were found to be significantly associated with anemia at a P-value of < 0 .05.

According to the binary logistic regression model, the odds of having anemia were 5.9 times [(AOR: 5.9 [(95% CI 1.04, 13.86)] higher among adults who took Zidovudine based ART regimen compared with TDF based regimen. Likewise, the odds of having anemia were almost 5 times higher among participants with CD4 count < 200 cells/mm^3^ compared with participants with CD4 count ≥ 500 cells/mm^3^ [(AOR: 4.8;( 95% CI 1.14,12.42)]. Two times more likelihood of anemia was demonstrated by participants who had inadequate dietary diversity compared with their counterparts [(AOR: 2.2; (95% CI 1.15, 4.26)]. Female sex [(AOR: 1.9; (95% CI 1.06, 3.69)] and educational status that is unable to read and write [(AOR: 3.2; (95% CI 1.24, 8.40)] were also significant predictors of anemia among HIV/AIDS infected adults on ART (Table 2).

**Table 2:** Bivariable and multivariable analysis of factors for anemia among HIV+ patients on ART at Debere Tabor Hospital, North West Ethiopia, 2017 (n = 365)

Variables	Anemia		COR (95% CI)	AOR (95% CI)
	Anemic	Non Anemic		
Sex				
Female	56	83	1.6 (1.01, 2.44)*	1.9 (1.06, 3.69)*
Male	68	158	1	1
Educational status				
Unable to read and write	62	59	3.3 (1.62, 6.79)*	3.2 (1.24, 8.40)*
Able to read and write	17	28	1.9 (.804, 4.559)	1.6 (.53, 4.67)
Primary school	19	72	0.832 (.373, 1.858)	1.1 (.39, 2.86)
Secondary school	13	41	1.00 (.414, 2.416)	1.4 (.45, 4.22)
Diploma and above	13	41	1	1
WHO clinical stage				
Stage I	55	156	1	1
Stage II	21	49	1.2 (.67, 2.21)	0.7 (0.27, 1.90)
Stage III	29	22	3.7 (1.98, 7.05)*	1.2 (.31, 4.28)
Stage IV	19	14	3.85 (1.808, 8.195)*	0.64 (.122, 3.328)
ART regimens				
AZT/3TC/NVP	38	103	5.6 (1.46, 22.61)*	5.9 (1.04, 13.86)*
AZT/3TC/EFV	14	22	1.4 (0.54, 3.74)	1.3 (0.39, 4.49)
TDF/3TC/EFV	57	87	2.4 (0.79, 7.48)	1.6 (0.41, 6.51)
TDF/3TC/NVP	6	23	1	1
CD4 count (cells/mm^3^)				
< 200	35	19	5.6 (2.95, 10.79)*	4.8 (1.14, 12.42)*
200–350	22	37	1.8 (0.97, 3.39)	1.2 (0.33, 3.98)
351–500	20	41	1.5 (0.798, 2.800)	2.0 (0.82, 5.08)
> 500	47	144	1	1
Dietary diversity score				
Inadequate DDS	96	145	2.3 (1.39, 3.72)*	2.2 (1.15, 4.26)*
Adequate DDS	28	96	1	1

* indicates significant at a p value of < 0.05

### Discussion

A variety of hematologic abnormalities associated with HIV infection have been described in different studies. One of them stats that anemia is a major public health problem in HIV positive patients around the world, particularly in Sub-Saharan African, including Ethiopia.

The overall prevalence of anemia in this study was 34% which was in line with findings from Rwanda (29%) [16], Central Ethiopia (33%) [20], and northwest, Ethiopia (35%) [21]. The result was higher than that of studies done in eastern India (16.2%) [22] Ghana (23.8%) [23] and North eastern Nigeria (24.3%) [24]. However, this report was lower than those of other studies done in China (51.9%) [14] and Tanzania (77.4%) [25].The variation in the burden of anemia between the current and later study settings could be related to disparities in socio-demographic characteristics and immunity. For instance, most of the study population (40.9%) in China had CD4 count less than 200 cells/mm^3^, while in this study only 14.8% of the participants had CD4 count less than 200 cells/mm^3^. This may be an important biological implication to our finding in that the mean hemoglobin level increased with increasing CD4 count, and lower CD4 count was associated with an increased risk of anemia.

Patients with CD4 cells < 200 cells/mm^3^ were more likely to be anemic compared to CD4 count ≥ 500 cells/mm^3^. This is supported by studies done in Diredawa Town, Ethiopia [2], and Zewditu Memorial Hospital, Addis Ababa, Ethiopia [26]. This could be explained by the fact that deterioration in the formation of hemoglobin due to a disrupted erythropoiesis results from the release of inflammatory cytokines and a decreased production of hematopoietic growth factors coupled with mal-absorption and impaired recycling of iron substance secondary to HIV/AIDS. Further-more, CD4 cells decrease the immunity of patients and expose them to opportunistic infections that lead the deficiency of micronutrient like iron.

In this study, patients who took AZT based ART regimens were more likely to be anemic than those who took TDF-based regimens. This finding is supported by studies done in Ethiopia [20], Rwanda [27], and Iran [28]. This is due to the fact that AZT has a myelosuppressive effect that reduces globin mRNA synthesis and a greater negative impact on hematologic parameters compared with the TDF-based regimens [29, 30]. Furthermore, Zidovudine treatment is associated with the suppression of bone marrow leading to a low production of red blood cells and other types of blood cells in the bone marrow. A meta-analysis of data from randomized trials confirms that anemia is more common with zidovudine- than stavudine-base triple-ART drugs [29].

In this study, women were more likely to be anemic than men. This is supported by a study done at University of Gondar Hospital, Ethiopia [21]. However, in a study done in Nigeria, sex was no significantly associated with anemia although arithmetically the prevalence of anemia was higher in women than in men [31]. The probable explanation might be that women in childbearing years are particularly susceptible to iron-deficiency anemia because of blood loss from menstruation and the increased blood supply demands during pregnancy. Older adults may also have a greater risk of developing anemia because of poor diet and other medical conditions.

This study also identified that the educational status was a predictor of the occurrence of anima among the study subjects. Patients who were unable to read and write were more likely to suffer from anemia than clients with higher educational level. The finding is supported by studies done in China and Nigeria [14, 24]. The probable explanation might be that study subjects with higher educational status are less likely to have unhealthy eating patterns (e.g., drinking carbonated beverages and eating instant noodles) than subjects who are unable to read and write. Furthermore, a higher intake of carbonated drinks was associated with a lower intake of protein, vitamins, and minerals that decrease the absorption of iron [32].

This study also noted that the likelihood of having anemia was higher among HIV/AIDS patients on ART with inadequate diversified diet. The explanation might be that dietary diversity is essential for nutrient adequacy as there is no single food that may contains all of the essential nutrients that are needed for good optimal health and good nutritional status. Moreover, when an individual consumes different foods or food items that promote sufficient intake of essential micronutrients from the diet and the hemoglobin level is improved [33].

### Conclusion

This study illustrated that anemia is a moderate public health problem among HIV infected adults on ART at Debre Tabor hospital. ART regimen, CD4 count, dietary diversity, educational status and sex were significantly associated with anemia. Therefore, the government needs to focus on routine screening, proper treatment, and increasing productivity to improve dietary diversification.

## Limitations of the study

In this study serum ferritin was not used as an indicator to identify iron deficiency anemia, and the data was collected in the fasting session of Orthodox Christians.

## Abbreviations

ANRS: Amhara National Regional State
ART: antiretroviral therapy
AZT: azidothymidine
BMI: body mass index
DDS: dietary diversity scores
EFV: efavirenz
FMOH: Federal Ministry of Health
GPs: general practitioners
HAART: highly active antiretroviral therapy
HCT: hematocrit
Hg: hemoglobin
HIV: human immune deficiency virus
NVP: nevirapine
OIS: opportunistic infection
PLWHA: people living with HIV/AIDS
RBC: red blood cells
SPSS: Statistical Package for Social Science
3TC: lamivudine
WBC: white blood cells
WHO: World Health Organization
ZDV: zidovudine
